# Arboreal crops on the medieval Silk Road: Archaeobotanical studies at Tashbulak

**DOI:** 10.1371/journal.pone.0201409

**Published:** 2018-08-14

**Authors:** Robert N. Spengler, Farhod Maksudov, Elissa Bullion, Ann Merkle, Taylor Hermes, Michael Frachetti

**Affiliations:** 1 Archaeology Department, Max Planck Institute for the Science of Human History (Max-Planck-Institut für Menschheitsgeschichte), Jena, Germany; 2 Institute for Archaeological Research, Academy of Sciences, Tashkent, Uzbekistan; 3 Anthropology Department, Washington University in St. Louis, St. Louis, Missouri, United States of America; 4 Institute for Prehistoric and Protohistoric Archaeology, Christian-Albrechts-Universität zu Kiel, Kiel, Germany; Institute of Geographic Sciences and Natural Resources Research Chinese Academy of Sciences, CHINA

## Abstract

During the first millennium A.D., Central Asia was marked by broad networks of exchange and interaction, what many historians collectively refer to as the “Silk Road”. Much of this contact relied on high-elevation mountain valleys, often linking towns and caravanserais through alpine territories. This cultural exchange is thought to have reached a peak in the late first millennium A.D., and these exchange networks fostered the spread of domesticated plants and animals across Eurasia. However, few systematic studies have investigated the cultivated plants that spread along the trans-Eurasian exchange during this time. New archaeobotanical data from the archaeological site of Tashbulak (800–1100 A.D.) in the mountains of Uzbekistan is shedding some light on what crops were being grown and consumed in Central Asia during the medieval period. The archaeobotanical assemblage contains grains and legumes, as well as a wide variety of fruits and nuts, which were likely cultivated at lower elevations and transported to the site. In addition, a number of arboreal fruits may have been collected from the wild or represent cultivated version of species that once grew in the wild shrubby forests of the foothills of southern Central Asia in prehistory. This study examines the spread of crops, notably arboreal crops, across Eurasia and ties together several data sets in order to add to discussions of what plant cultivation looked like in the central region of the Silk Road.

## Introduction

The ‘Silk Road’ was an historically and archaeologically documented cultural phenomenon, characterized by gradually increasing interaction that connected communities in Central Asia to a larger social and economic sphere [1]. Scholars are increasingly exploring the broader process of exchange through Central Asia, including the study of corridors of diffusion from the third millennium B.C. onward and systematic exchange systems of the late first millennium B.C. onward, collectively constituting a wide range of goods and peoples [2, 3, 4, 5]. This cultural arena of interconnectivity tied certain Central Asian populations into a shared realm of commodity transfer, resulting in the spread of material goods over vast distances. Approaching the concept of the Silk Road, it is harder to pin the term down to one time period or cultural phase, especially seeing that scholars have been studying pre-Silk Road exchange in the archaeological record for decades [1, 6, 7, 3]. Furthermore, many of the most important routes of Eurasian exchange transected some of the highest mountain ranges in the world [8]; however, limited archaeological investigation has been undertaken outside the major urban centers at lower elevations [9]. Furthermore, there is still little scientific inquiry into what goods were actually moving along the historical trade routes [10]. We use archaeobotanical data to study what crops were actually consumed at these medieval towns and compare the data to other sites in order to explore the spread of domesticated plants across the ancient world. We suggest that orchards and vineyards around the oasis cities of Central Asia, such as Bukhara, Khiva, Loulan, and Samarkand, provided cultivated goods for merchants and travelers, who in turn carried those fruits and grains along a nodal network and ultimately across two continents. In this article, we synthesize medieval-period botanical data and present a systematic study of botanical remains recovered from anthropogenic sediments from Tashbulak, Uzbekistan (A.D. 800–1100). We also suggest that most of the fruit crops identified in this archaeobotanical assemblage were carried to the site by merchants from lower elevations, based on the fact that many of these trees cannot grow at high elevations. By pulling together these diverse data sets and contrasting them with historical sources, we argue that arboreal crops were a prominent part of the economy across Central Asia during this period and that certain crops dispersed across Eurasia through Central Asia.

### Historical sources

The archaeobotanical data that we present in this paper fit into an historical context; in order to interpret these data, we must understand what the historical sources tell us regarding the cultivation of crops in medieval Central Asia. Early literary accounts from Central Asia attest to the fruits of medieval urban markets and the commerce routes that spread them. For example, Abu Hamid al-Andalusi al-Gharnati traveled in Khorezm from 1130–1155 and described cities, villages, farmsteads, and fortresses. He also stated that there were “fruits, the like of which I have not seen in any of the other countries I have visited” ([11]:88); specifically describing melons, dates, red and white grapes, apples, pears, pomegranates, and watermelons. About a century earlier, in 988, Ibn Hawqal described Khorezm as a “fertile country, producing many kinds of grain and fruit” ([11]:177). Zahiruddin Muhammad Babur, wrote that “[g]rapes, melons, apples, and pomegranates, all fruits, indeed, are good in Samarkand; two are famous, its apple and its şāhibī (grape)” ([12]:77). Nuruddin Muhammad Jahangir (1569–1627) repeatedly stated that farmers near Samarkand grew exceptionally sweet apricots, peaches, melons, and apples, as well as rice, millet, and wheat. He specifically noted that “[o]n one tray they brought many kinds of fruit—Kārīz melons, melons from Badakhshan and Kabul, grapes from Samarkand and Badakhshan, apples from Samarkand, Kashmir, Kabul, and from Jalalabad, which is a dependency of Kabul, and pineapples, a fruit that comes from the European ports” ([13]: Vol. 1:73). Al-Jahiz supposedly wrote a preserved late first millennium pamphlet on trade, which focuses on luxury goods coming into the Abbasid capital of Baghdad [14]. He extensively discusses fresh and dried fruits as one of the most prominent trade goods. A cache of Sogdian documents recovered from the Mugh (ca. 600–800 A.D.) citadel discuss economic transactions, notably the trade of large amounts of barley grains and wine [15]. Contemporaneous documents from the region also note the importance of the exchange of dried fruits and even suggest that some people paid their taxes in dried fruits and nuts [15]. The archaeobotanical data collaborate these historical texts by illustrating that people were moving fruits and nuts and that a diversity of cultivated foods was used in the cuisines of medieval Inner Asia.

### Comparative archaeobotanical data

**Fig 1:**
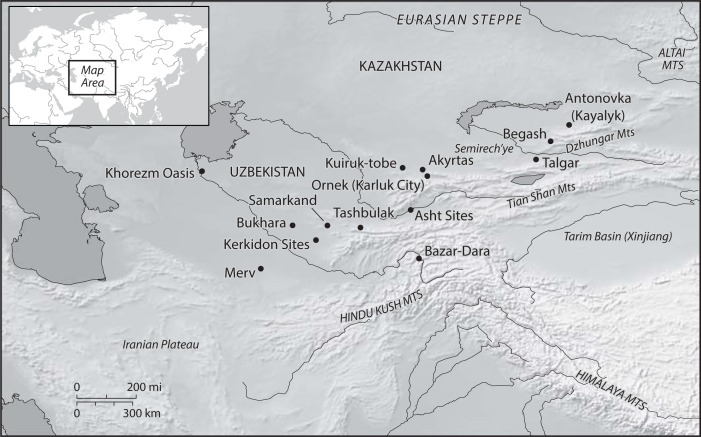
A map of Central Asia showing the location of medieval archaeological sites that have reports of preserved archaeobotanical material (carbonized and/or desiccated).

**Fig 2:**
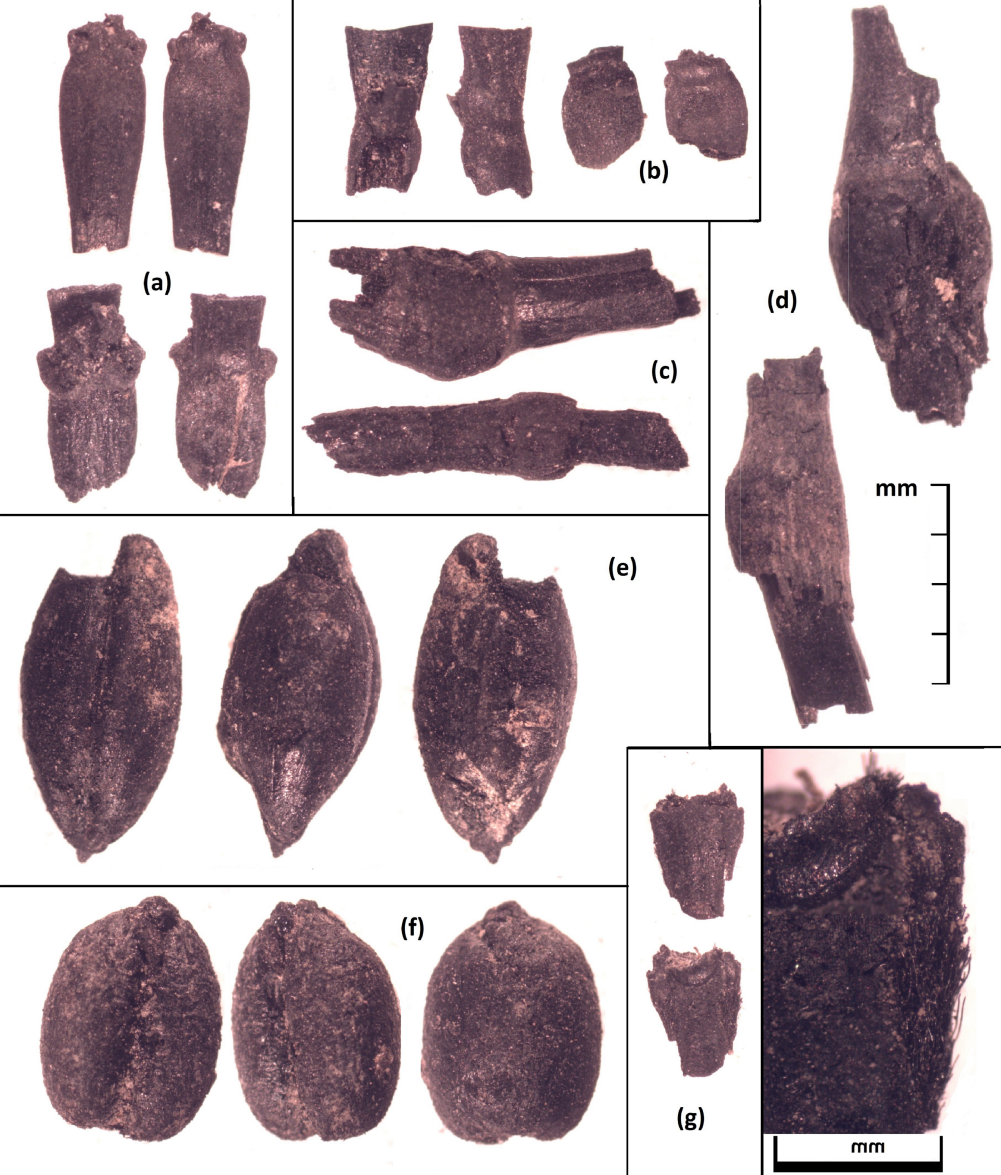
**Cereal Grains from the Tashbulak site**: a) an example of a hexaploid wheat rachis on the top and a tetraploid wheat rachis on the bottom; b) hexaploid wheat rachises; c) and d) culm nodes of a grass species that is roughly the size of a typical cereal plant; e) three views of a barley grain; f) three views of a compact free-threshing wheat grain; and g) a barley rachis with a close-up of the hairs along the margin.

Archaeological studies complement the historical sources by illustrating how diverse economic strategies across Eurasia were at this time, surveys have identified the presence of cities, towns, and small farmsteads [9, 16, 17]. Stable isotope analyses of bone collagen demonstrated highly diverse diets between regions that were narrowly circumscribed within communities across this varied cultural landscape [18]. Examining previous studies of botanical remains from medieval contexts helps us understand how our new data fits into a broader Eurasian dispersal of domesticated trees. These findings are, in most cases, unpublished or hard to access outside Central Asia. While direct dates, photos, and descriptions of the identification criteria are lacking for most of these reports, they merit further consideration. In eastern Kazakhstan, a small archaeobotanical assemblage was collected at the fortified town of Talgar (ca. 700–1200 A.D.; Fig 1); it contained remains of barley (*Hordeum vulgare*), both naked and hulled forms, broomcorn millet (*Panicum miliaceum*), and compact wheat (*Triticum aestivum*) grains, as well as wild seeds, notably *Polygonum* and *Onopordum acanthium* [19]. Another small study at the same site was conducted by Miller [20]; the 2.4L of sediment contained free-threshing wheat and barley grains, a possible oat (*Avena* sp.) grain, and numerous wild seeds and dung fragments. Two additional small samples from the following season (1.0 and 1.5L of sediment) also contained wheat, barley, a possible foxtail millet grain (*Setaria* sp.), and wild seeds–*Chenopodium*, *Convulvolus*, *Cyperaceae*, *Trifolium*, *Trigonella*, *Hypericum*, *Plantago*, and *Galium* [20]. Also in eastern Kazakhstan, three flotation samples (24L of sediment total) from the medieval occupation (1220–1420) at Begash (Fig 1; [2, 21]) were analyzed, containing 45 broomcorn and 11 foxtail millet grains, as well as 1,217 identified wild seeds or seed fragments representing 21 distinct categories of seeds. At Antonovka (Fig 1), 22 flotation samples and 382 liters of sediment from thirteenth century layers were floated [19]. The assemblage consisted of 1,500 wild herbaceous seeds, including *Chenopodium*, *Oenothera*, *Lithospermum arvense*, *Rubus*, and *Anchusa arvensis*, and 178 domesticated grains and 80 legumes. Of the grains, 119 were of a highly compact form of wheat, the remainder came from hulled barley and broomcorn millet, and among the legumes, 69 were peas (*Pisum sativum*), the rest were lentils (*Lens culinaris*).

Archaeobotanical studies in the Talas Valley of southern Kazakhstan were conducted on sediments from a fortified structure that was excavated at Akyrtas (ca. 900–1200 A.D.) and a mosque at Ornek (Karluk; ca. 900–1200 A.D.), both of which contained domesticated grains of wheat, naked and hulled barley, and broomcorn millet, unspecified legumes, as well as wild seeds of *Chenopodium*, *Lithospermum arvense*, and *Polygonum*. Grape pips (*Vitis vinifera*; n = 3) and apple/pear seeds (n = 7) were also recovered from Ornek ([19]:99). The nearby site of Kuiruk-tobe provided hand-picked desiccated grape pips and possible watermelon seeds (*Citrella lanatus*) [19]. In flotation samples from Djuvan-tobe (ca. 600–900 A.D.), Bashtannik [22] identified 178 carbonized specimens: 65 barley grains; 61 broomcorn millet grains; 24 free-threshing bread wheat grains; 14 peas; 4 lentils; and grape pips. The most interesting discovery from the seventh century occupation layers was a single rice (*Oryza sativa*) grain. He also recovered a single rice grain from sediments from Karaspan-tobe (ca. 300–500 A.D.), as well as a purported plum pit (*Prunus* sp.), grape pips, peas, and millet, barley, and wheat grains.

At several sites in Ferghana, a Soviet excavation team dissolved unfired mudbrick in water and hand collected large carbonized seeds from within [23]. The mudbricks came from fifth to seventh century layers in the Osh Region at the border of Kyrgyzstan. Barley, both hulled and naked forms, was the most prominent grain from Kuyuk Tepe and Tudai Kalon. Compact and lax-eared forms of wheat were recovered from the sites of Kuyuk Tepe, Munchak Tepe (compact), and Tudai Kalon. Broomcorn millet grains were also recovered from all three of these sites and seeds from a small-seeded form of lentils were recovered from Kuyuk Tepe and Munchak Tepe. Peas and an unspecified vetch (*Vicia* sp.) were reported from Kuyuk Tepe. Rice grains were also reported from Munchak Tepe, on the Sokh alluvial fan near the Syr Darya River, as were grape pips, peach and apricot stones, and melon and unspecified cucurbit seeds ([24, 23]:176). *Papaver* sp., although likely wild, and cotton seeds (*Gossypium* sp.) were recovered from Tudai Kalon and Kuyuk Tepe, as well as a slightly latter (ca. 600-800A.D.) site in the same region, called Ak Tepe [23]. An abundance of capper seeds (*Capparis spinosa*) was recovered from a burial at Damkul Cemetery, also near Kerkidon [23]. Grapes represent the most prevalent fruit found at Kerkidon, and at the nearby site of Settlement 5a a possible winery was excavated. Wineries have been reported from medieval sites across southern Central Asia [25]. Gorbunova [23] also references a study from the 1970s, where a series of occupation sites and burial grounds in the Asht Region of northern Tajikistan were excavated, hand-picked seeds at those sites supposedly included melons (*Cucumis melo*), watermelons, unspecified nuts, peaches (*Prunus persica*), apricots (*P*. *armeniaca*), cherries (*Prunus* sp.), apples (*Malus pumila* or *M*. *sieversii*), and almonds (*P*. *dulcis*). An even more interesting series of botanical discoveries came from the 1933 excavations at the fortified citadel at Mugh (ca. 600–800 A.D.) in northern Tajikistan, which is usually associated with Sogdians. Among the handpicked remains of cultivated plants, the excavators noted cherry, peach, apricot, grape, apple, and cotton seeds, grains of hulled barley, free-threshing wheat, broomcorn millet, a large amount of walnut (*Juglans regia*) and almond shells, and legumes that they claim are fava beans (*Vicia faba*) [15, 26]. They also claimed to have grains of cultivated *Echinochloa crus galli*, a weed or loosely domesticated grain that is sometimes allowed to grow or is planted in fields in Tajikistan and Uzbekistan. Yakubov [15] also referred to finds of plum, cherry, peach, and apricot pits, as well as apple and grape seeds from contemporaneous sites in the region around Mugh. Furthermore, Lunina [25] noted the remains of fruits in a well at the southern Sogdian city of Nesef, dating between the eighth and twelfth centuries; these finds include apple, watermelon, cucurbits, and grape seeds, peach and apricot pits, and walnut and almond shells.

One of the largest archaeobotanical study conducted in Central Asia consists of hand-collected plant remains from Bazar-Dara (cliff-market; nearly 4,000masl), Tajikistan [27]. The ancient high-elevation town would have been supplied by caravans from lower elevation farming centers and is roughly contemporaneous with Tashbulak. Bubnova [27] theorized that there would have been steady supply routes carrying fruits, nuts, legumes, and grains to the mining communities at these higher elevations [27]. There are also high-elevation remains of ancient water mills for grain grinding in the Aksu River valley. Among the wide variety of cultivated fruits and grains recovered at the site, the excavators identified wheat and barley as well as one possible rye (*Secale cereale*) grain [27]. Interestingly, they also report to have found rice, as well as *Vicia*, peas, and lentils. The range of fruits that Bubnova [27] identified from the site is unparalleled at any other archaeological site in Eurasia, while there are no direct dates on the material, and neither photographs nor morphological descriptions were made, she claimed that the project botanists identified melon (13,112 seeds) and watermelon seeds (n = 246), mulberry seeds (*Morus* sp.), walnut shells (7,474), other nut shells, apricot and peach pits, almond shells (n = 10,315), apple (n = 975) and pear (n = 10,782) seeds, cherry pits, as well as pits of what she called cherry plums (with over 9,000 pits), pistachio (*Pistacia vera*) shells, grape pips (supposedly recovering 52,805 from house 2 at the site and over 5,000 from the other contexts), and barberry seeds (*Berberis* sp.). She also claims to have found a few fruits and nuts that have never been identified in archaeological sites in Central Asia before, but the finds are not impossible to believe given the extent of the trans-Eurasian exchange during this time period, including date pits (*Phoenix d*actylifera), hazelnut shells (*Corylus* cf. *avellana*), persimmon seeds (*Diospyros* sp.), and most astonishing of all, one coconut shell (*Cocos nucifera*) and some form of unspecified cucurbit seed, possibly *Lagenaria siceraria*. It is important to note that there were later occupations at the site and some of these finds could have been recovered out of context.

Merv (Fig 1) was briefly, during the twelfth century A.D., one of the largest cities in the world and arguably the most strategic ‘fueling station’ along the Silk Road. In 1992 and 1993, M. Nesbitt conducted extensive analysis on 1,074 liters of sediment from early medieval layers, comprising 100 samples and 62 discrete deposits. They published a synthesis of the major finds in Herrmann and Kurbansakhatov [28]. Of the 62 contexts that were sampled, 37 contained domesticated plant remains–ubiquities (number of contexts in which the given plant category appears) were provided for the 11 cultivated species recovered. The most ubiquitous was cotton, appearing in 78% of the contexts, followed by hulled barley (46%) and free-threshing bread wheat (38%); broomcorn millet was only represented in 3% of the contexts. Legumes were represented by lentils (24%) and peas (8%). Fruit and nut remains from the site included, grapes (8%), hackberries (*Celtis* sp.; 8%), melons (3%), peaches (3%), and almonds (3%) [28]. Moving further west, Brite et al. [29] brought together a list of Soviet-period (mostly 1960-1970s) mentions of domesticated crops in archaeological reports. They noted the presence of grapes, millet, and barley as far back as the fifth century B.C., and peaches, Russian olives (*Elaeagnus angustifolia*), and melons possibly appearing sometime between the fourth century B.C. and the fourth century A.D. They also noted grass peas (*Lathyrus sativa*), possible oats (*Avena sativa*), and possible alfalfa (*Medicago sativa*) by the fourth to fifth centuries A.D. They provided references from an archaeological report from 1966 claiming sorghum (*Sorghum bicolor*) by the sixth to eight centuries A.D. and rice by the seventh to eighth centuries A.D. Collectively, these data illustrate that cultivation was a prominent part of the medieval Central Asia economy; they also show that fruit orchards were maintained across a large geographic area and crop varieties were dispersed across Eurasia during this period.

## Materials and methods

Excavations at the archaeological site of Tashbulak were conducted as part of the ARQ (Archaeological Research of the Qarakhanids) project, in collaboration with the Institute of Archaeology in Samarkand, a branch of the Academy of Sciences of Uzbekistan. Appropriate excavation permits were obtained through the institute and the project was codirected by Michael Frachetti and Farhod Maksudov. Tashbulak is a medieval town located at roughly 2,200 masl in the Malguzar range of eastern Uzbekistan. At such a high-elevation, the site is above the growing ecocline for many of the fruit trees that were identified at the site. Hence, this site would have been, at least in part, supplied by lower elevation farming communities through trade and supply routes, with grains and legumes cultivated locally. A 2x1 meter (roughly 2 meters deep) trench was cut into a midden deposit (identified through magnetometry) in the center of the town, next to what has been interpreted as the ancient market bazaar (South Unit in Table 1). Flotation samples from this midden, in association with material from a nearby larger excavation unit (North Unit), have provided remains of carbonized and preserved fruits and grains. This archaeobotanical study consists of 22 sediment samples, comprising sediments from 13 contexts at the site. The volume of each sample varied greatly, from roughly 1 liter to as much as 37.5L (FS25). In total, 513 liters of soil were floated, 223L of which is reported in this paper. Spengler floated these samples in the field using a bucket method [30, 31, 32]. Light fraction was collected down to 0.355mm, although systematic sample sorting was only conducted down to 0.5mm. Heavy fractions were collected down to 2.0mm, after several attempts to sieve the sediments through a 1.0mm sieve failed, due to heavy gravel content in the colluvium. Heavy fractions were sorted in the field using a 5x hand lens. All carbonized organic material and cultural artifacts were collected. Cultural artifacts, including beads, glass, metal fragments (most notably a fragment of silver foil), sherds, and slag, were handed over to the project for cataloging with other excavated artifacts. The macrobotanical remains were identified using a comparative collection of Central Asian seeds and several identification keys. The identifiable seeds and fruit parts were quantified and recorded in the data table, S1 Table. The archaeobotanical assemblage will be cataloged and stored in the archaeological archival collections at the Max Planck Institute for the Science of Human History in Jena, Germany, in association with the Archaeobotanical Laboratories, under the directorship of Robert Spengler. In addition, a digital assemblage of photos of key specimens will be made available online through robertnspengler.com after publication.

**Table 1:** Total seed counts, density, ubiquity, and liters of sediment from two excavation units at Tashbulak.

**Grains and Legumes**	# of Samples	Liters of Sediment	Wheat (Free-threshing)	Barley (Hulled/ Naked)	Peas	Chickpea	**Total Grains/ Legumes**	Cerealia
North Unit	16	123.5	44	4	1	1	50	51
South Unit	6	100.0	103	40	3	6	152	56
**Total**	22	223.5	147	44	4	7	202	107
Density (Seeds/L)	North		0.4	0.0	0.0	0.0	0.4	0.4
	South		1.0	0.4	0.0	0.1	1.5	0.6
	**Total**		0.6	0.2	0.0	0.0	0.9	0.5
Ubiquity (% of samples)	North		63	13	6	6	69	63
	South		100	67	33	50	100	100
	**Total**		77	27	14	18	77	73
**Wild or Cultivated Fruits**	# of Samples	Cappers	Cherry	Hack-berries	Rose Hip	Russian Olive	Sea Buck-thorn	Walnut
North Unit	16	2	1	1	24	1	0	12
South Unit	6	2	5	6	26	0	7	14
**Total**	22	4	6	7	50	0	7	26
Density (Seeds/L)	North	0.0	0.0	0.0	0.2	0.0	0.0	0.1
	South	0.0	0.1	0.1	0.3	0.0	0.1	0.1
	**Total**	0.0	0.0	0.0	0.2	0.0	0.0	0.1
Ubiquity (% of samples)	North	6	6	6	44	6	0	40
	South	17	67	83	100	0	17	67
	**Total**	9	23	27	59	5	5	45
**Cultivated Fruits**	Apple	Apricot	Grape	Melon	Peach	Pistachio	**Wild Seeds**	**Total Seeds**
North Unit	0	2	5	0	0	1	1135	1254
South Unit	13	7	31	6	1	4	4510	4803
**Total**	13	9	36	6	1	5	5645	6057
Density (Seeds/L)	0.0	0.0	0.0	0.0	0.0	0.0	9.2	10.2
	0.1	0.1	0.3	0.1	0.0	0.0	45.1	48.0
	0.1	0.0	0.2	0.0	0.0	0.0	25.3	27.1
Ubiquity (% of samples)	0	13	19	0	0	6	94	100
	50	50	67	17	17	17	100	100
	14	23	32	5	5	9	95	100

## Results

The archaeobotanical assemblage consists of 6,057 identified carbonized seeds and fruit parts (S1 Table), as well as 371 unidentifiable seed fragments. Of the identifiable specimens, 204 were from domesticated field crops and 196 were from fruits or nuts (grape and melon seeds are included in this category), which may or may not have been cultivated. The remaining 5,657 seeds and fruit parts come from small wild herbaceous plants or in a few cases, such as *Potentilla*, possibly short woody plants. An additional 107 Cerealia fragments were also identified, but not counted in the totals due to the inability to determine Minimum Number Individual (MNI) estimates on these fragments. The Cerealia category was used when a grain fragment was too damaged to differentiate between wheat and barley. In discussions of abundance and density, Cerealia fragments are considered separately, because several fragments could have come from one seed. In addition, 58 free-threshing hexaploid wheat rachises, 8 barley rachises (Fig 2), 61 culm nodes (likely from cereal straw), and 6 grape pedicles were recovered, but none of this material was added to the final counts in order to avoid artificially doubling the quantities for several of these key categories.

Free-threshing hexaploid wheat (*Triticum aestivum* ssp. *aestivum*; n = 147) and hulled barley (*Hordeum vulgare* var. *vulgare*; n = 44) represent the domesticated grain crops from the site. Two possible long narrow durum or macaroni wheat (*T*. *turgidum*) grains and a few rachises from a tetraploid wheat were also recovered. Both wheat and barley were recovered at Tashbulak in association with their rachises, allowing us to categorize the wheat (Fig 2A). Additionally, two legumes were identified, peas (n = 4) and chickpeas (*Cicer arientinum*; n = 7). In addition to the grains and legumes, six melon (*Cucumis* cf. *melo*) seeds from FS24 represent the only other annual crop in the assemblage. Melons are an important part of Central Asian cuisine today, and thousands of landraces are maintained across Eurasia. Of the economically significant arboreal species recovered from the site, at least four of them appear to represent non-native species–peaches, apricots (*Prunus* cf. *armeniaca*), grapes, and apples (*Malus/Pyrus*). While there are wild Malus and *Prunus* species native to the area, the specimens of *Malus/Pyrus*, *P*. *persica*, and *P*. cf. *armeniaca* from the site all appear to represent domesticated forms. Many of the apricot pits are fragmentary and we cannot confidently rule out other *Prunus* taxa. The remaining eight economically significant fruits and nuts recovered from the site all represent species that are native to the foothill region of Uzbekistan, and while it seems likely that several of them were cultivated in orchards; it is also possible that some of them were collected from the wild. The native species of fruit and nuts that appear in the Tashbulak assemblage include: cappers; cherries (*Prunus* subgenus *Padus*); hackberries; pistachios; sea buckthorn (*Hippophae rhamnoides*); rose hips (*Rosa* sp.); Russian olive; and walnuts (Table 1 and S1 Table). These specimens may have come from cultivated, maintained, or wild plants; although, it seems likely that at least some of them were cultivated, notably the pistachios, walnuts, and Russian olives.

## Discussion

Exchange routes of the Silk Road ran through the Asian mountain foothills, and plants moved along these routes in unison with people, textiles, metal goods, ceramics, and a wide range of commodities [9, 33]. The fruits and nuts from Tashbulak represent the first study of the plants that were passing through these high-elevation routes. The data we present in this paper link the mountain routes of Eurasia, seeing that similar species have been identified at early archaeological sites as far south as Pakistan and as far north as Xinjiang. At archaeological sites in northern Pakistan and Kashmir after 2000 B.C., macrobotanical remains of almonds, apricots, grapes, hackberries (*Celtis caucasica*), and peaches have been identified [34, 35, 5]. Likewise, second millennium B.C. finds in desiccated burials from Xinjiang include seeds of grapes, apricots, broomcorn millet, Job’s tears (*Coix lacryma-jobi*), naked barley, peaches, Russian olives, and walnuts [36].

### Grains

While wheat and barley have been identified across Central Asia dating back several millennia prior to occupation at Tashbulak, the specific varieties of cereal recovered from the site are much larger than forms identified at earlier sites. Furthermore, tetraploid wheat has never been recovered from Central Asia before; although, Watson [37] suggested that they spread with the Islamic conquests across Eurasia. The dearth of archaeobotanical data for tetraploid wheats in Central Asia prevents us from approaching the topic of the crop’s relationship with Islam. However, tetraploid wheats are an important crop in the southern Islamic world today. In addition to the typical macaroni wheats (*Triticum turgidum* ssp. *durum*), farmers in southern Central Asia and across the Iranian Plateau and Mesopotamia historically grew Khorezm wheat (*T*. *turgidum* ssp. *turanicum*), a variety typically attributed to the broader northern Iranian region, and Persian wheat (*T*. *turgidum* ssp. *carthlicum*), a lesser known morphotype grown across southwest Asia in the past. We did not quantify the number of hexaploid verses tetraploid wheat rachises, due to the damaged state of many of the specimen; however, there appear to only be a few tetraploid examples. The rachises and culm nodes also suggest that the crops were grown near the site and that the chaff was processed close to the site; this conclusion is further attested by the presence of barley chaff impressions on a few of the sherd fragments, showing that it was used as binder. Interestingly, neither of the East Asian millets (*Panicum miliaceum* and *Setaria italica*) were identified in the assemblage, despite the importance of these crops across Central Asia for millennia prior; nor is rice present, despite its importance in local cuisines today.

### Grapes

**Fig 3:**
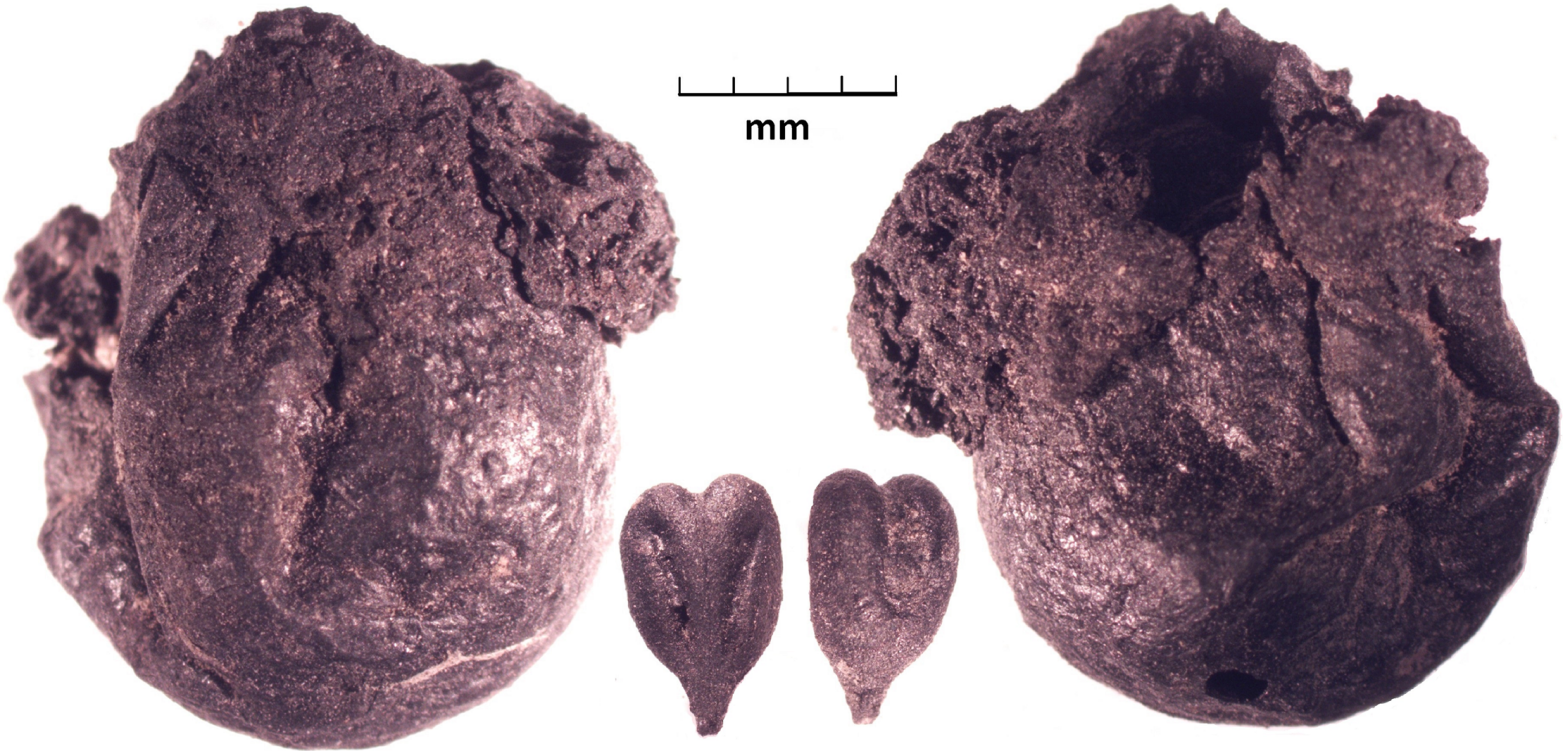
Front and back view of a whole preserved grape with a ventral and dorsal view of a grape pip, all from Tashbulak.

Wine is closely associated with Sogdian merchants in historical texts, especially at the oasis cities that span the region of Xinjiang and northern Qinghai in western China. ‘Uighur’ wine production techniques are still practiced in some of these towns, especially around Kashgar, and viticulture can be traced back in this region to the mid-first millennium B.C., as is evidenced by illustrations of grape vines on a textile from the Sampula Cemetery in Turfan, an actual 116-cm-long vine preserved in a tomb from the Yanghai Cemetery [36], and pips from Tuzusai in eastern Kazakhstan [21, 38]. Wine production in western China became increasingly popular during the Tang Dynasty as new varieties of grapes, notably the infamous “mares’ nipples”, spread into the region with Central Asian immigrants along the Silk Road [39, 33]. The grape pips, pedicles, and even a whole carbonized grape (Fig 3) from Tashbulak further attest to the important role that grapes and presumably wine played into the Islamic era of Central Asia.

### Apples

Recent genetic research has shown that the true ancestors of our modern apple, *Malus sieversii*, are from southeastern Kazakhstan [40, 41, 42]. However, the Silk Road is directly responsible for the modern large-fruited, sweet, and heavy-fruiting varieties of apples that we cultivate today. After the original cultivation of these trees in Central Asia, “[p]eople then took the domesticated apples westwards along the great trade routes known as the Silk Route, where they came into contact with other wild apples, such as *Malus baccata* (L.) Borkh. in Siberia, *Malus orientalis* Uglitz. in the Caucasus, and *Malus sylvestris* Mill. in Europe” ([40]:59). The Silk Road led to genetic bottlenecking, hybridization, and further human selection; later, grafting and cloning allowed for the fixation of traits. Interestingly, there have been almost no published finds of apple seeds from pre-medieval sites in Central Asia, with the exception of one possible *Malus* seed recovered at Gonur in Turkmenistan (2500–1700 cal B.C.) [43] and one possible seed from Tuzusai [38]. However, by the Classical period domesticated apples had already made their way to Europe. It is not possible to determine if the apple seeds from Tashbulak are from a wild or cultivated form, and it must also be noted that there are a few other close apple relatives that originated in Central Asia and have seeds that overlap with *Malus* morphologically, notably quince (*Cydonia oblonga*) and pear (*Pyrus* spp.).

### Peaches and apricots

**Fig 4:**
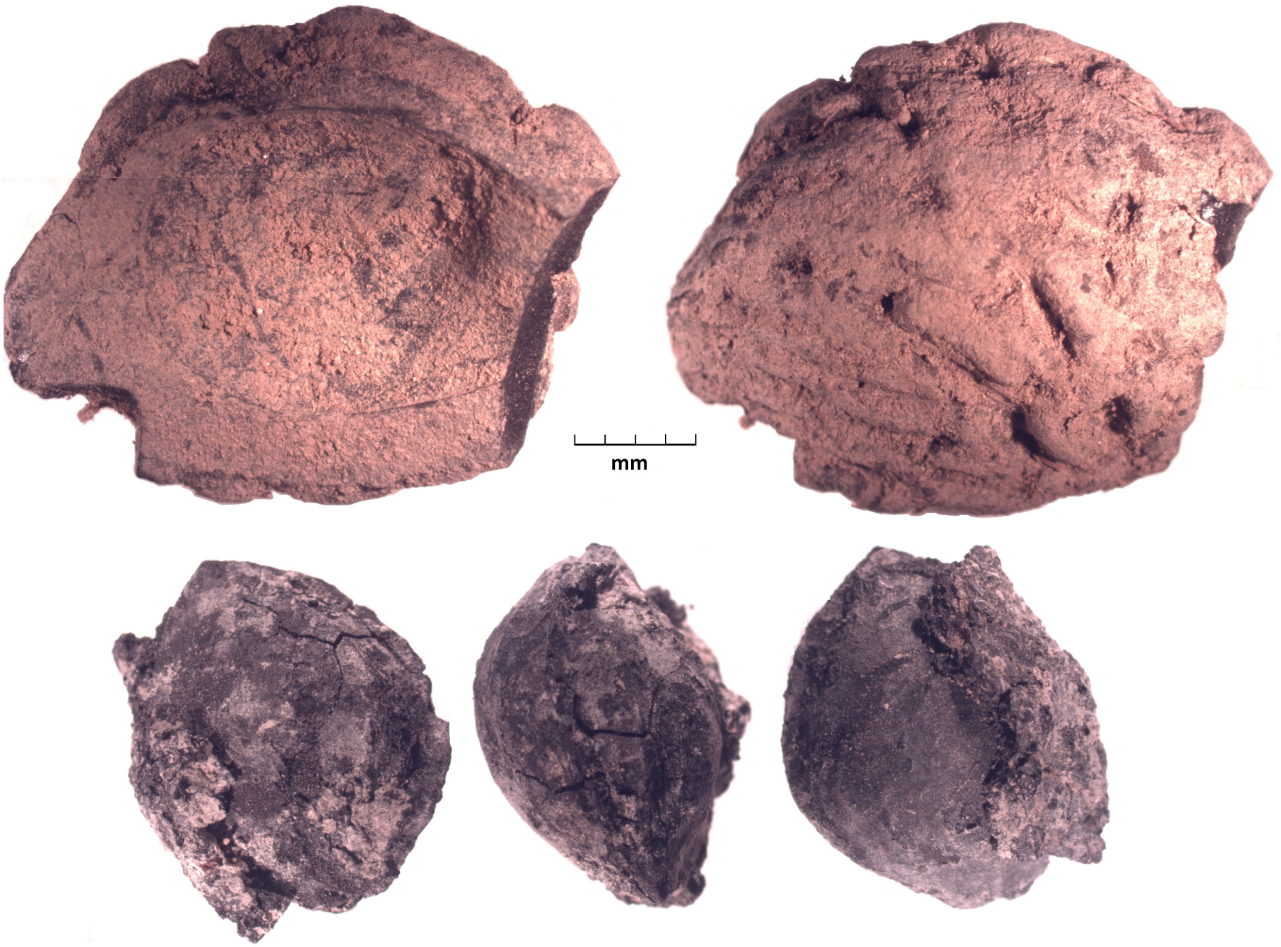
Three views of a carbonized apricot pit on the bottom, with fruit flesh still adhered to the stone, and two views of half of a peach pit, top, both from Tashbulak.

Tashbulak provided remains of several species of rosaceous fruits, especially from the *Prunus* genus (Fig 4). Recent archaeobotanical data seems to place the original cultivation of the peach in the marshes of the Yangtze River in Zhejiang, China [44, 45]; however, they are present in the Mediterranean by the mid-first millennium B.C. [46, 47]. The pits of these fruits have been recovered from burials at the Sampula Cemetery (400–100 B.C.) [36]. While this data seems to support a dispersal through Central Asia, early evidence for peaches have also been recovered in the Indus Valley, northern Pakistan, and Kashmir by 2000 B.C. [34, 48, 5]. The peach tree has a deep-seeded tradition in East Asian cultures and by the Tang Dynasty was clearly an important commodity along the Silk Road. In fact, the legendary “Golden Peaches of Samarkand” [39] were introduced to Emperor Taizong, as a gift from Sogdians. There is an ongoing debate over the origin and dispersal of the apricot, which has a long historical tradition in the Caucuses and other parts of the Old World; however, the oldest evidence for the fruit comes from eastern China [45]. There are a number of close wild relatives of the cultivated apricot that possess large fruits and even a few other relatives, such as *P*. *spinose*, that have unclear historical dispersals; therefore, the Tashbulak apricot stones require further study.

### Melons

**Fig 5:**
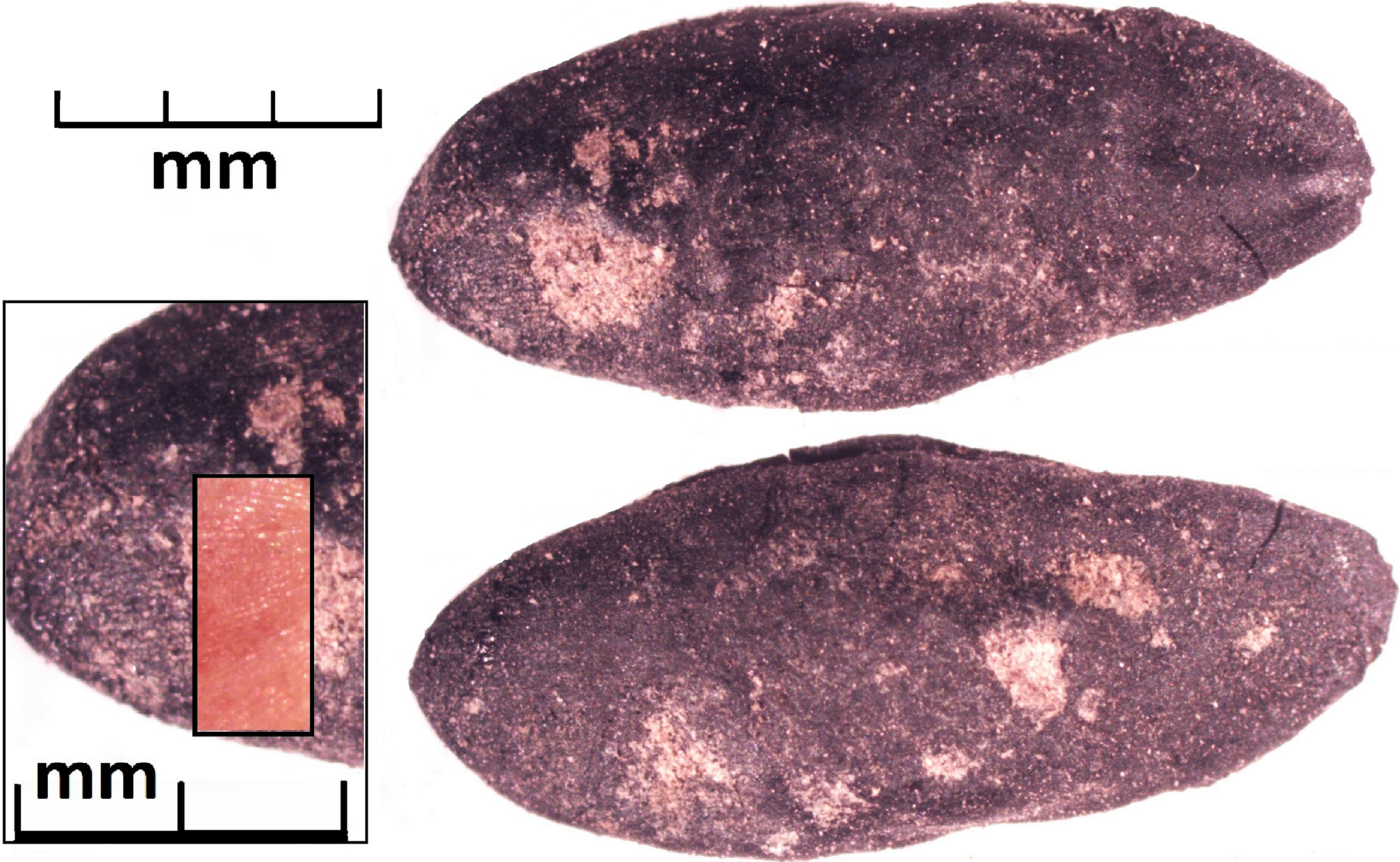
Two side views of one carbonized melon seed from Tashbulak, with an inset photo of a close-up of the surface striations and a double inset photo of a modern melon seed with similar surface striations.

Six melon (*Cucumis* cf. *melo*) seeds, all from one sample, were also recovered from Tashbulak (Fig 5). While the Asian melons (*Cucumis melo* ssp. *inodorus*) hold a deeply rooted tradition along the Silk Road, other *Cucumis* crops have historically been cultivated in Central Asia, including the cucumber (*Cucumis sativus*) and Persian melon (*C*. *melo* ssp. *cantalupensis*) [49]. There are thousands of regionally specific ancient melon landraces across Central Asia, with one of the most famous being the Hami melon from Xinjiang. Due largely to the fact that the seeds are unlikely to be carbonized and preserve, there is a dearth of data on their origins and spread [49]; recent genetic studies have proposed several possible origins for the cucurbit.

### Pistachio and walnut

The prominent nuts of historical Central Asian bazaars included almonds, the roasted pits of peaches and apricots, pistachios, and walnuts. The nutshells recovered at Tashbulak include all of these except almonds. *Pistacia vera* is the progenitor of our modern commercial pistachio. While *P*. *terebinthus* and *P*. *acuminate* were well known in the Classical world, *P*. *vera* originated in the foothills of Central Asia and is a later introduction to Europe. There are only a handful of early archaeobotanical remains of this economically significant species, including reports from the second millennium B.C. sites of Tepe Yahya in Iran and Djarkutan and Gonur in southern Central Asia [43]. The shells of this species were also identified at Sarazm, Uzbekistan [50] showing that there is a long history of collecting this nut along the Zerafshan foothills. The king or Carpathian walnut (i.e. *regia*) has a large range of distribution spanning from Eastern Europe to the foothills of western China. Wild forests of this species of walnut are native to the mountain slopes of Central Asia, and walnut forests are still present at mid-range altitudes (1,500–2,800masl) in the Ferghana Valley. Recent genetic work suggests that disparate populations of the walnut hybridized as the trees were exchanged through Central Asia and eventually Europe [51].

### Other fruit

There is no reason to believe that the cherry pits from Tashbulak, which are rather small and have a poorly defined ridge, were cultivated, especially seeing that there are dozens of wild species that grow in Eurasia and at least two that grow near the site today. The archaeobotanical pits do not resemble *Prunus avium* or *P*. *cerasus*. Like the cherries, many of the other fruits and nuts in the assemblage may either represent wild foraging, management of wild fruit-bearing trees, or even early forms of arboriculture based on native trees that would eventually become domesticated. Both sea buckthorn and Russian olives are currently cultivated and were domesticated in Central Asia. *E*. *angustifolia*, *E*. *angustata*, and *E*. *oxycarpa* are currently cultivated or maintained in semi-wild stands in the foothills, but also grow wild across Central Asia. Rose seeds (*Rosa* sp.) were also recovered at Tashbulak, and three species of wild roses grow immediately around the site today, *R*. *canina*, *R*. *ecae*, and *R*. *maracandica*. Furthermore, hackberry seeds (*Celtis caucasica*) are represented in the assemblage, a species that has been collected from the wild in this region since at least the third millennium B.C. [50].

## Conclusions

The forests that once covered the Central Asian foothills contained several economically significant fruit and nut species, which were an important part of the economy, notably wild walnut, almonds, and pistachios. The archaeobotanical specimens may represent a gradual process of maintenance and eventual cultivation of wild species in the region or the hybridization of various populations of the same species, as demonstrated through population genetics studies of apples and walnuts [40, 51]. The dispersal of these crops along the Silk Road played a significant role in their domestication and eventual adoption by farmers across Europe and Asia. Several other fruits in the assemblage represent domesticated crops that originated thousands of kilometers away. Notably, apples were originally domesticated in the Tien Shan further north in Central Asia, grapes were domesticated in western southwest Asia or the eastern Mediterranean, and peaches and apricots appear to have been domesticated in the Yangtze River valley of eastern China.

The Tashbulak assemblage provides the first systematically collected and recorded data point for most of these crops in Central Asia. It is also important to acknowledge that many of these fruits were not cultivated at or around the site. Night frosts at these high elevations continue through the spring and would damage or destroy the flowers for most of these arboreal crops. While some of the grains are grown at high elevations today, fruit trees are restricted to mid- or low elevations in the foothills. Therefore, these fruits were transported to the site from lower elevations, either dried or fresh, when in season. Furthermore, historical and art historical sources emphasize how highly regarded certain fruit varieties were during the past millennium in Central Asia and how readily people spread them between population centers.

The comparative data we present in this paper illustrate the role that the trans-Eurasian exchange routes played in spreading cultivated crops across Eurasia. In contrasting our new data with the reports from other contemporaneous sites across Central Asia, we can discuss what role these crops played in the broader economy and when certain crops first appeared at these towns. This study is particularly important for understand the dispersal of arboreal crops, which have received limited attention from scholars in the past. Fruit orchards were cultivated around every medieval village or urban center across Central Asia, and fruits were carried to high-elevation towns. Ultimately, this trade in fruits and nuts lead to the dispersal of these crops across Europe and Asia.

## Supporting information

S1 Table Full archaeobotanical data table for the Tashbulak project. (PDF)
