# Correction: Arboreal crops on the medieval Silk Road: Archaeobotanical studies at Tashbulak

**DOI:** 10.1371/journal.pone.0204582

**Published:** 2018-09-20

**Authors:** Robert N. Spengler, Farhod Maksudov, Elissa Bullion, Ann Merkle, Taylor Hermes, Michael Frachetti

The affiliation for the sixth author is incorrect. Michael Frachetti is not affiliated with #2 but with #3 Anthropology Department, Washington University in St. Louis, St. Louis, Missouri, United States of America
