# Supplementary material for: Arboreal crops on the medieval Silk Road: Archaeobotanical studies at Tashbulak
Source: PLoS One. 2018 Aug 14;13(8):e0201409. doi: 10.1371/journal.pone.0201409 (PMC6091944; doi:10.1371/journal.pone.0201409)
Supplement: S1 Table — (PDF) [file pone.0201409.s001.pdf]

S1_Table

Tashbulak, Uzbekistan							Grain Parts *Not in Totals					Domestic Grains and Legumes					Fruits and Nuts															Amaranthaceae		Asteraceae			Boragenaceae	Brassicaceae		Caryophyllaceae		Convolvulaceae	Cyperaceae	Fabaceae				Malvaceae	Poaceae					Polygonaceae		Papaveraceae	Plantaginaceae	Rosaceae	Rubiaceae	Scrophulariaceae	Solanaceae		Thymelaeaceae		Unidentified	Unidentifiable	Totals
Site	Trench	Context	Grid Coordinates	Sample #	Volume of Soil Floated (Liters)	Wood (Fragments > 2.00 mm) Ct.	Wheat Rachis	Barley Rachis	Cerealia	Culm Node	Grape Pedicle	*Hordeum vulgare* var. *vulgare*	*Triticum aestivum* (Free-threshing)	*Triticum turgidum* (Free-threshing)	*Pisum sativum*	*Cicer arietinum*	*Capparis spinosa*	*Celtis caucasica*	*Cornus* sp.	*Cucumis* (Cf. *melo*)	*Elaeagnus augustifolia*	*Hippophae rhamnoides*	*Juglans regia*	*Malus/Pyrus*	*Prunus* (Cf. *armeniaca*)	*Prunus persica*	*Prunus* (Subgenus *Padus*)	*Pistachia vera*	*Rosa* sp.	*Vitis vinifera*	Nutshell	Cheno-ams	*Chenopodium* spp.	Asteraceae	Achene Seed Only	Asteraceae Type A	*Lithospermum arvense*	*Thlapsi* Type	*Lepidium/Capsella* Type	Caryophyllaceae	*Vaccaria* (Cf. *hispanica*)	*Convolvulus* sp.	Cyperaceae	Fabaceae	*Medicago* sp.	*Trigonella* sp.	Cf. *Trifolium*	*Malva* sp.	Poaceae	Poaceae Type A	Pooid	*Setaria* (Wild)	*Stipa* Type	Polygonaceae	*Polygonum* spp.	*Papaver* sp. (Presumably Wild)	*Plantago* sp.	*Potentilla/Fragaria*	*Galium* sp.	*Veronica* sp.	Solanaceae	*Hyoscyamus niger*	*Thymelaea* sp.	Cf. *Daphne*	Unidentified Seeds	Unidentifiable Seed Fragments	Totals without Unidentifiable Fragments
Tashbuak	North Unit	6	W19-G8	FS1	7.5	134	1		5			3	3																1		1							5										2												12	1						28
		6	W19-F8	FS2	8.5	148	1		2	1			2																				5															2							3			5					1		1	3	19
		6	W19-G9	FS3	8	99			1				1																			1	1															4							1			1	2						3		14
		5	X19-E3	FS4	7.5	5																	1								2																																				3
		7	X19-D3	FS5	5	41			1																				1			3	6									1						2	2						8	2		6	2						2	4	39
		9	X19-E3	FS6	7	42	1						1																				13										1		6			1			1				16			36		1						9	76
		6	X19-G2	FS7	6.5	89																										1	1								1														4			2									9
		9	X19-G2	FS8	7.5	231			4				1		1								4									2	2																1						6			1									18
		11	X19-F1	FS9	7.5	311							2										1								1	1	1																						3											1	9
		12	X19-F2	FS10	7.5	360	2		3				5												1				1	1																									3				2							1	13
		12	X19-F2	FS11	1	65																																										1							1			1									3
		13	X19-D3	FS12	23	N/C	2		28				18			1	2				1		2		1				11		2	4	76			2					4	2	8		2	2		2	4						57			188	1	3					3	18	396
		11	W19-G8	FS13	7.5	101			3				4																1				2																									7									14
		11	W19-G8	FS17	7	49			3	2			7					1					3							2	2		2															1							7				1							7	26
		13	X19-E3	FS18	5	51			1	1																			4	2		2	1							1			4		3	10									58			262		1					1	10	349
		13	X19-E3	FS19	7.5	100						1											1				1	1	5			2	35							1			2		4	2									29			153					1			4	238
				Subtotal	123.5	1826	7	0	51	4	0	4	44	0	1	1	2	1	0	0	1	0	12	0	2	0	1	1	24	5	8	16	145	0	0	2	0	5	0	2	5	3	15	0	15	14	0	15	7	0	1	0	0	0	196	2	0	662	8	17	1	0	2	0	10	57	1254
	South Unit	L4	N20-D8/9	FS24	22.5	N/C	9	1	14	2	4	17	34				2	2		6			3	7			1	4	5	2		225	491		3	4	3	8	37	11	59	5	19		3	2	35	10	3	10	1		6		337	5	1	135	56	80	4			4	20	75	1660
		L5	N20-D8	FS25	37.5	N/C	7	2	34	6		12	21		1	4		1						5	5	1			13	27	5	133	464		2		7	13	25	14	91	13	20		9	9	42	9	9	30	3		5	4	393		2	133	53	97	3		1	1	12	93	1785
		L6	N20-D8	FS26	15	N/C	24		3	29		8	12						1				2	1					2	1		2	89	1		2	1		2	1	2	8	5	1	2			3	1	11	2		3		83	2		78	17						5	64	348
		L7	N20-D8	FS31	14	N/C	2	3	2	16	1	3	17			1		1				7	6		1		2		1	1	5	3	61			2		2	4		4	4	9			7		4	12		1		2		36			45	11	17		3			1	41	273
		L8	N20-D8	FS32	7.5	365	2		2		1		3					1	1						1		1		4			2	202			2	5	8		2	2		2			3		7				1			20			28	3	30		5			2	12	335
		L9	N20-D9	FS37	3.5	N/C	7	3	1	4			16	2	2	1		1					3				1		1		7	10	251			1	3						3					6	2		1				15	1		19	41	11				2	2	29	402
				Subtotal	100	365	51	9	56	57	6	40	103	2	3	6	2	6	2	6	0	7	14	13	7	1	5	4	26	31	17	375	1558	1	5	11	19	31	68	28	158	30	58	1	14	21	77	39	27	51	8	1	16	4	884	8	3	438	181	235	7	8	1	7	42	314	4803
				Totals	223.5	2191	58	9	107	61	6	44	147	2	4	7	4	7	2	6	1	7	26	13	9	1	6	5	50	36	25	391	1703	1	5	13	19	36	68	30	163	33	73	1	29	35	77	54	34	51	9	1	16	4	1080	10	3	1100	189	252	8	8	3	7	52	371	6057
